# The Impact of Immune Checkpoint Inhibition on the Risk of Radiation Necrosis Following Stereotactic Radiotherapy for Metastatic Brain Cancer

**DOI:** 10.7759/cureus.51381

**Published:** 2023-12-31

**Authors:** Ben Royal-Preyra

**Affiliations:** 1 Radiation Oncology, Centre Hospitalier Affilié Universitaire Regional, Trois-Rivières, CAN

**Keywords:** treatment of brain metastases, cancer immunotherapy, radiation necrosis, checkpoint inhibitors, stereotactic radiotherapy

## Abstract

Purpose/objective

Forty percent of cancer patients develop brain metastases (BM) and are often treated with stereotactic radiation (SRS/SRT). Checkpoint inhibitor (CI) use is suspected of increasing the risk of radiation necrosis (RN). Our aim is to determine whether treatment with CI is associated with an increased risk of RN in BM patients treated with SRS/SRT.

Methods

We retrospectively identified the medical records of BM patients treated with SRS/SRT between 1/2017 and 12/2021 using an institutional database. RN was defined by MRI imaging read by neuroradiologists and/or surgical pathology. V12GY of patients with and without RN was compared using the Mann-Whitney test. The chi-square test was used to see if RN was associated with CI use, histology, particular CI agent used, > 1 course SRS/SRT, SRS/SRT dose, chemotherapy, whole brain radiotherapy (WBRT), age, or sex.

Results

Two hundred and fifty-nine patients treated with 455 courses of SRS/SRT were analyzed. The most common primary histologies were lung 56% (N=146), breast 14% (N= 37), melanoma 9% (N=24), and renal cancer 7% (N=18). A total of 53.8% (N = no. of patients) were treated with CI. The overall rate of any RN was 21.8% (N=27) in the CI group compared to 14.8% (N=141) in the non-CI group (p=0.174). Mean V12Gy was 15.525 cc and 9.419 cc in patients with and without RN (p=0.02768). Mean number of SRS/SRT courses was 2 and 1.53 for patients with and without RN, and >1 course of SRS/SRT was a predictor of RN (p <0.01). Other features analyzed were not significant.

Conclusion

RN was higher in the BM patients treated with SRS/SRT receiving CI compared to non-CI patients (21.8%, N=27, versus 14.6%, N= 16), but failed to reach statistical significance. V12Gy and > 1 course of SRS/SRT was associated with RN. Caution should be taken in treating patients with SRS/SRT and CI there might be an increased risk of RN.

## Introduction

Almost two million Americans were diagnosed with cancer in 2022 [1], 40% of these patients will develop brain metastases (BM) [2], and up to 26% will be treated with stereotactic radiosurgery or fractionated radiotherapy (SRS/SRT) [3] due to published literature showing excellent local control (LC) [4,5]. Since the FDA approval of ipilimumab in 2011, checkpoint inhibitor (CI) use has increased, and in 2020, up to 44% of US cancer patients were eligible for treatment with CI at some point during their disease course [6]. Radiotherapy and CI are theorized to act synergistically and might lead to greater LC when used in combination [7]. The ablative doses of radiotherapy used in SRS/SRT can enhance the antitumor activity of the immune system and, in some cases, even lead to abscopal-like responses in the brain [7]. The available data suggest that combining CI and SRS/SRT improves outcomes compared to radiotherapy alone and that concomitant treatment is more effective than sequential use [8]. A meta-analysis by Lehrer et al. that included 534 patients and 1570 BM showed improved LC and overall survival (OS) with concurrent SRS and immune CI therapy and reported only a 5.3% risk of radiation necrosis (RN) [9]. There is a strong rationale in radiation oncology to study the potential benefits of combined CI and SRS/SRT and to characterize the possible increased toxicity risks. The most important toxicity from cranial irradiation is RN [10]. The risk of RNs following cranial radiation therapy (RT) ranges from 1.5% to 28%, with most studies reporting rates of 5-10% [10]. RN usually occurs 6-15 months post-RT, and potential treatment options include steroids, surgery, Avastin®, and hyperbaric oxygen [10]. RN management is complicated because it is hard to define and cannot be reliably differentiated from tumor progression using conventional imaging techniques [11]. Approximately 50% of RN cases are asymptomatic, radiologic findings only and can be safely observed with subsequent imaging, whereas symptomatic cases often require treatment with medications and, sometimes, neurosurgical intervention [11]. Published literature of single fraction treatments has found that the volume of normal brain receiving 12 Gy or more (V12Gy) is associated with RN risk and that a V12 Gy greater than 5-10 cc leads to a risk of greater than 10% RN [10-12]. One seminal publication by Minniti et al. found that a V12Gy greater than 10.9 cm^3^ had a risk of RN of 47% [10]. Milano et al. found more moderate rates of RN of 15% with V12 Gy greater than 10 cc and 20% with V12 Gy greater than 15 cc [13]. Various authors have reported on the features that impact the risk of RN in patients treated with SRS/SRT, such as target volume [14,15], V12Gy [10-13], male sex [15], total dose and fractionation [16], prior cranial radiation [17], chemotherapy [17], tumor location and histology [17]. The N0574 trial by Brown et al. randomized 213 patients with 1-3 BM to treatment with SRS +/- whole brain radiotherapy (WBRT) and found no difference between the two groups in terms of RN (2.9% with SRS alone versus 4.5% with SRS + WBRT, p=0.72) [18]. Similarly, the phase 3 N107C trial by Brown et al. randomized patients treated with surgical resection of one BM to post-op SRS versus whole brain RT and found reassuringly low rates of grade 2 or higher RN of 4% for SRS and 0% for WBRT [19]. It is important to note that neither of these studies included patients treated with immunotherapy or patients treated with multiple courses of radiotherapy. The available published literature is mixed on whether CI use increases the risk of RN [20-30].

This study aims to determine whether treatment with CI is associated with increased rates of RN in BM patients treated with SRS/SRT. These data can be used to determine whether a subset of patients is at particularly high risk of developing RN if treated with SRS/SRT and potentially impact treatment decision-making, such as altering the choice of dose/fractionation or CI agent used.

## Materials and methods

Using an institutional database, we conducted a retrospective review of all patients with metastatic cancer treated for BM with SRS/SRT between 1/2017 and 12/2021 at the Beth Israel Deaconess Medical Center. After institutional research ethics board approval (2017P000635), we reviewed patients’ charts and recorded demographic, histological, staging, radiation, systemic therapy data, clinical notes and imaging, and treatment toxicity details. Specific information recorded included age at first SRS/SRT, sex, primary histology of each patient’s cancer, the number of SRS/SRT courses each patient received, dose and fractionation of each SRS/SRT treatment, whether the patient received WBRT, the dose and fractionation of WBRT, whether patients were diagnosed with RN and whether this was a radiological or pathologic diagnosis, the time from the first course of SRS/SRT to RN diagnosis, any RN treatments received, the V12Gy of each course of SRS/SRT, the date of last follow up, length of follow up for each patient, death date (if available), and the systemic therapy agent used and the number of courses of chemotherapy or immunotherapy received was recorded for each patient. The primary endpoint examined was RN (radiologic and/or pathologic). RN was defined by MRI imaging read by American Board of Radiology certified neuroradiologists and/or surgical pathology (if available) interpreted by pathologists at our institution. The V12Gy of patients who developed RN versus those who did not was compared using the Mann-Whitney U test. The chi-square test was used to compare the difference in RN rates between patients treated with and without CI, the difference in RN rates for each specific CI agent used, the RN rates for patients treated with 1 course of SRS/SRT versus > 1 course of SRS/SRT, the RN rates between different SRS/SRT doses/fractionations, the RN rates between primary histologies, the RN rates for patients aged <65 versus aged >65 years, the RN rates in male versus female patients, and the RN rates for patients treated with and without chemotherapy. Significance was set at p = 0.05 for both the Mann-Whitney U test and the chi-square test.

## Results

We identified 282 patients with BM treated with Cyberknife® (CK) SRS/SRT (Accuray Inc., Sunnyvale, California, USA) between 1/2017 and 12/2021, 8.2% (N=23) patients were excluded due to receiving SRS/SRT at an outside institution, having a primary CNS malignancy, or having no available follow-up documentation. A total of 259 patients treated with 445 courses of SRS/SRT were included in the analysis. The median length of follow-up time for all patients was 11.31 months. The median time from the 1st course of SRS/SRT to RN was 13.68 months. Thirty-one percent (N=81) of patients were still living at the end of the study period, 51% (N=133) were deceased, and 18% (N=47) had unknown vital status. The overall rate of any RN for the entire cohort was 16.4% (N=43). A total of 72.1% (N=189) of patients did not develop RN, 3.8% (N=10) had equivocal radiologic RN findings, and 7.6% (N=20) had unknown RN status. The median age of the cohort was 68.8 years. Forty-four percent (N=116) of the patients were biologically male, and 56% (N=146) were female. The most common primary malignancies were lung 56% (N=146), breast 14% (N=37), melanoma 9% (N=24), and renal 7% (N=18). Table 1 shows the full breakdown of primary malignancies identified.

**Table 1:** Primary histologies of patients treated with stereotactic radiosurgery

Primary Histology	% (N)
Lung	56% (146)
Breast	14% (37)
Melanoma	9% (24)
Renal	7% (18)
Other/Not specified	4% (11)
Colorectal	4% (10)
More than one	3% (7)
Uterine	<1% (2)
Ovarian	<1% (2)
Choriocarcinoma	<1% (2)
Esophagus	<1% (2)

The rate of RN among the four most common primary histologies was compared using chi-square statistic (see Table 2). No statistically significant difference was found. The chi-square statistic was 4.2019, p=0.2405.

**Table 2:** Rates of radiation necrosis among primary histologies

Histology	Total Patients	Radiation Necrosis (N)	Rate of Radiation Necrosis (%)
Lung	146	19	7.68%
Breast	37	9	4.11%
Melanoma	24	6	4%
Renal	18	3	6%

A total of 53.8% (N=141) of patients received CI at some point in their disease course, and 45% (N=118) did not. CI use was unknown for 1.1% (N=3) of patients. For patients for whom both CI use and RN status were known, the rate of any RN was 21.8% (N=27) in the CI group compared to 14.8% (N=16) in the non-CI group (p=0.1736). Table 3 shows the different CI patients received.

**Table 3:** Immune checkpoint inhibitors received by patients

Checkpoint Inhibitor Used	% of Patients (N)
Pembrolizumab	28.2% (N=74)
Nivolumab	11.1% (N=29)
More than one (most commonly: Ipilimumab/Nivolumab)	9.2% (N=24)
Durvalumab	1.9% (N=5)
Atezolizumab	1.5% (N=4)
Other	1.5% (N=4)
Ipilimumab alone	0.3% (N=1)

The rates of RN among patients treated with the most common CI were compared using chi-square statistic. No statistically significant difference was found (see Table 4). The chi-square statistic was 1.1735, p=0.556134.

**Table 4:** Rate of radiation necrosis and checkpoint inhibitor agent used

Checkpoint Inhibitor Agent	#Patients without Radiation Necrosis	# Patients with Radiation Necrosis	Total # Patients	% Patients with Radiation Necrosis
Pembrolizumab	64	10	74	13.5%
Nivolumab	23	6	29	20.7%
More than one	19	5	24	21.0%

To compare whether an RN risk difference exists between various doses of SRS/SRT, we compared the rates of RN for patients treated with only one course of SRS/SRT for one metastasis/cavity (see Table 5).

**Table 5:** Radiation necrosis rates for various stereotactic radiosurgery/radiotherapy doses for patients treated with one course

Radiation Dose	# Patients with Radiation Necrosis	# Patients without Radiation Necrosis	Total # Patients	% Patients with Radiation Necrosis
22 Gy/1	4	19	23	17.4%
20/1	1	4	5	20%
24/3	4	23	27	14.8%
27/3	1	5	6	16.7%
25/5	0	11	11	0%
30/5	1	4	5	20%
27.5/5	0	1	1	0%
20/5	1	0	1	100%
Total patients	12	67	79	

The overall rate of RNs in this cohort was 15.2% (N= 12). The rate of RN between SRS/SRT doses was not statistically different. Chi square statistic: 8.011818, p=0.331552. The mean cumulative V12 Gy (all SRS courses combined) for the 24 patients with RN, treated with single fraction SRS, and with V12 data available was 15.525 cc. The mean cumulative V12Gy (all SRS courses combined) for the 125 patients without RN and V12 data available was 9.419 cc. The mean V12Gy for patients with RN and without RN was compared using a two-sample Mann-Whitney U test (two-tailed), resulting in a statistically significant result with a Z statistic of =2.2018, p=0.02768. The mean number of SRS/SRT courses was 2 for patients with RNs and 1.53 for patients without RNs. Forty percent (N=105) of patients were treated with >1 course of SRS/SRT, and 60.6% (N=157) were not. A total of 78.3% (N=203) of patients received chemotherapy at some point during their disease course, 19.7% (N=51) did not, and 1.9% (N=5) had unknown chemotherapy use status. In sum, 15.4% (N=40) of patients received WBRT, 84.2% (N=218) did not, and for 0.4% (N=1) of patients WBRT status was unknown. Overall, 51.3% (N=133) of patients were ≥65 years old and 49.8% (N=129) were <65 years old. The chi-square test was used to see if RN rates differed with chemotherapy use, treatment with > 1 course of SRS/SRT, treatment with WBRT, age >65, and sex (see Table 6). Only treatment with >1 course of SRS/SRT was found to be statistically significant (p < 0.01).

**Table 6:** Chi-square statistic for the variables analyzed for an association with radiation necrosis

Variable	# Patients with Radiation Necrosis	# Patients without Radiation Necrosis	Chi-square Statistic (CSS), p-value (p)
Chemotherapy	36	146	CSS: 0.6749, p = 0.411361
No Chemotherapy	7	41	
1 course of stereotactic radiosurgery	18	121	CSS: 7.1629, p = 0.007443
>1 course of stereotactic radiosurgery	25	68	
Whole Brain Radiotherapy	6	29	CSS: 0.0529, p = 0.818148
No Whole Brain Radiotherapy	37	160	
Age < 65 years	25	91	CSS: 1.3988, p = 0.236926
Age > 65 years	18	98	
Male	19	85	CSS: 0.0088, p = 0.925331
Female	24	104	

Of the 36 patients with RN and management details available, 75% (N=27) received dexamethasone, 42% (N=15) underwent surgery, 22% (N=8) received Avastin, and 25% (N=9) received no treatment due to asymptomatic necrosis/radiological changes only.

## Discussion

Our data are consistent with the published literature showing RN rates of 2.9-29% in patients with BM treated with immunotherapy and SRS/SRT [16-31]. Two publications looked at patients treated similarly to ours and found RN rates of 15% and 6-17% respectively [32,33]. The available published literature is mixed regarding whether treatment with immune CI inhibitors increases the risk of RN in BM patients treated with SRS/SRT. A meta-analysis published in 2020 that included 2,365, mainly melanoma, patients receiving ipilimumab +/- PD-1/PD-L1 directed therapy, showed improved LC and OS without increased rates of RN [20]. A recently published multicenter study involving groups in the United States, Canada, and Italy included 55 renal cell patients with 395 BM treated with CI and SRS/SRT and found no increased risk of RN with CI use [21]. There are also many case series of patients with BM treated with SRS/SRT and CI resulting in improved LC and OS without an apparent increase in toxicity [22-27]. In contrast to this, other studies have shown a significant increase in RN in BM patients treated with SRS/SRS and CI. For example, one publication found that RN occurs 2.4 times more frequently in melanoma BM patients treated with SRS and CI than SRS alone (16% vs. 6.5%, P=0.065) and that melanoma BM patients had greater rates of RN than non-small cell lung cancer patients [8]. Data presented at the American Society for Radiation Oncology (ASTRO) annual scientific meeting in 2017 found that CI use increased the rate of RNs from 8% to 19%, but this failed to reach statistical significance [29]. A 2018 retrospective paper that included 480 patients showed increased symptomatic RN in BM patients treated with anti-CTLA-4 agents, but not PD-1 agents [30]. In our study, treatment with CI was associated with an approximately 50% relative risk increase of RN (21.8%, N=27, versus 14.6%, N= 16), but this failed to reach statistical significance. Due to the significant limitations of this study, including the retrospective design, the lack of data on the timing of CI use in relation to SRS/SRT, the 8.2% of patients lost to follow-up, and a limited follow-up length which could reduce our ability to capture all RN events, caution should be taken in treating patients with CI and SRS/SRT as this might be associated with an increased risk of RN. Our study is consistent with the published literature publications showing an association between V12Gy [10-13] and prior courses of RT [17] and RN. However, paradoxically, our data were also consistent with the literature showing a lack of association between RN and treatment with WBRT [18,19]. This could potentially be explained by patients with more advanced intracranial disease or poorer performance status receiving WBRT and not living long enough to develop RN.

Despite the weaknesses present in this study, there is a relatively large sample size of 259 patients which should be adequate to show a clinically meaningful difference in the rates of RN for the features analyzed. Features of interest that were not analyzed in this study include whether concurrent versus sequential CI use leads to different RN rates or whether the rates of asymptomatic RN (e.g. radiologic only) differ with CI use. Possible further avenues of research include analyzing the dosimetric parameters other than V12Gy that impact RN in patients treated with SRS/SRT [34], the use of imaging modalities other than gadolinium-enhanced MRI to assess RN [35], and the use of machine learning to help distinguish RN from tumor progression [36].

## Conclusions

These data suggest that caution must be taken in treating patients with SRS/SRT and CI. CI use was associated with a 50% relative risk increase of any RN in this study, although this was not statistically significant. The lack of statistical significance could be due to methodological limitations rather than a lack of association between treatment with SRS/SR, CI, and RN and clinicians should be careful when weighing the potential benefits of combining CI and SRS/SRT against the risks of increased toxicity. The finding that repeated courses of radiotherapy lead to increased toxicity is consistent with the published literature. It is becoming more relevant as expanded systemic therapy options lead to more prolonged patient survival and more opportunities for re-treatment. Larger prospective trials are needed to quantify the dosimetric parameters other than V12Gy associated with RN to enable clinicians to estimate the risk of toxicity with multiple treatments with SRS/SRT.
